# The perspectives of patients, nursing students and supervisors on “the caring–learning space” – a synthesis of and further abstracton of previous studies

**DOI:** 10.1080/17482631.2023.2172796

**Published:** 2023-02-06

**Authors:** Hanna Holst, Lise-Lotte Ozolins, David Brunt, Ulrica Hörberg

**Affiliations:** Department of Health and Caring Sciences, Linnaeus University, Växjö, Sweden

**Keywords:** Caring, clinical practice, learning, lifeworld, phenomenology

## Abstract

The aim was to describe and gain a greater understanding of the phenomenon “caring-learning space” based on the perspectives of patients, nursing students, and supervisors in clinical practice contexts.

A general structure of the phenomenon “caring-learning space” was created based on essential structures from five empirical studies. The analysis is based on a reflective lifeworld research approach (RLR).

The “caring-learning” space shows itself in terms of interpersonal relationships between patients, students, supervisors, and other actors in the care environment. It is first when the learning space is accepted as a part of caring, that a “caring-learning space” is created. A flexibility and a receptivity are seen where the learning is allowed to become visible and be integrated in the caring processes when caring and learning to interact.

A caring-learning space is established by the patient being the focal point, but also a co-creator in caring as well as learning regardless of her/his health status. This entails that the ”caring-learning space” exists when patients, students, supervisors and other healthcare professionals interact based on their ability and role in the space.

## Background

Nursing students’ learning in clinical practice that includes the patient perspective is a complex phenomenon. This complexity encompasses both being cared for as a patient and learning in a caring environment as a student nurse where caring and learning take place simultaneously. A key challenge in nursing education is thus to provide the tools, opportunities and environment for students to develop caring skills based on compassion and craftmanship (Adamson, 2018). Furthermore, previous research has shown that student learning in clinical practice has mostly been described from the perspective of students and supervisors (Pålsson et al., 2021;Williams et al., 2021) and only rarely from the patient perspective (Suikkala et al., 2018).

Recent research reveals that nursing students’ learning in clinical practice needs to create space for the learning activities that involve the development of compassionate care, proposing that learning should be based on the patients’ narratives and experiences (Adamson & Dewar, 2015;Rosser et al., 2019) and further supported by reflection (Kolderstam et al., 2021). Supporting students’ learning in clinical practice aims at enabling the students to be active in their learning to care for the patients. This support can be guided by a reflective approach to support nursing students in taking responsibility in the care for the patient and in preparing for their future professional role as a carer. Moreover, the importance of a supportive clinical learning environment provided by staff, supervisors and peer learning has been emphasized (Vuckovic et al., 2021). This is in line withCant et al. (2021) who described that the educational atmosphere is associated with the supervisory relationship, the leadership of the ward and the premises where nursing takes place rather than the performance of clinical nursing tasks. Furthermore, they propose the need for greater investment in nursing clinicians in teaching, presenting a positive and welcoming learning culture on the ward, for enabling appropriate resources, for enabling evidence-based clinical practices and the development of a working relationship between the education and healthcare staff. Learning to care is thus complex and requires didactic strategies to support students’ learning in clinical practice and to prepare the nursing students for their future career as nurses.

One potentially useful didactic strategy for the student to gain the ability to care in this complex situation is to use the patient’s own narrative as a starting point. This approach, which is based on a lifeworld perspective and emphasizes the importance of reflection, has been described by Ekebergh (2007; 2009;Ekebergh, 2011). This procedure has been utilized in a few studies where the patient perspective of being cared for by nursing students has been described, suggesting that caring science based on lifeworld-led reflection supports both the students’ learning and the patients´ health processes (Andersson et al., 2020;Eskilsson et al., 2015). The lifeworld theory was introduced byHusserl (1936/1970 who described it as the world we experience and that every person has an unique lifeworld. Everyday experiences are taken for granted according to the lifeworld theory and this natural attitude is unreflective and can be problematized by reflection (Husserl, 1936/1970). This theory has been used to develop lifeworld-led caring and learning; the former entails understanding the perspective of the patients in caring, who are seen as experts in themselves concerning their well-being, health and illness. Similarily, lifeworld-led learning entails understanding the perspective of the students in learning to care, who are understood as experts in themselves concerning their learning. Furthermore, the patient is at the centre of the care, and the student is at the centre of the learning, and the focus is on supporting patients’ well-being and alleviating suffering, and developing the students in learning to care (Dahlberg & Ekebergh, 2008).

Andersson et al. (2020) and Ekebergh and Lindberg (2020) describe the importance of paying attention to the interaction of caring and learning in clinical practice in order to strengthen the caring process and sensitivity towards the patient situation. This is in line with the findings concerning the learning space created in interpersonal interactions between patient, pairs of students and supervisor (Holst et al., 2017b).

The environment for learning and caring needs to be seen as one (intertwined) where the learning and caring strategies interact with a caring-learning approach. It can thus be important to intertwine caring and learning into one new phenomenon to gain a new understanding instead of seeing them as two separate phenomena, and also to explore the perceptions of the actors in this context. The aim of the present study was thus to describe and gain a greater understanding of the phenomenon “caring-learning space” based on the perspectives of patients, nursing students, and supervisors in clinical practice contexts.

## Method

Five empirical studies (Table 1), based on a lifeworld approach and focusing on different perspectives of caring and learning, have previously been performed and have been chosen as the data material for a new analysis. A further abstraction and synthesis of the findings from these studies has been carried out to achieve the aim of this study, resulting in a general structure. Firstly, the method used and its methodological foundation to form a synthesis/general structure is described, followed by how the new analysis of the five studies in this article was performed to create the general structure. The empirical foundation, a table and a short summary of the five studies are presented in order to understand the clinical practice concept. Furthermore, the general structure is described in the results section.

**Table I.** Overview of the five empirical studies.

Study	I	II	III	IIII	V	I-V
Participants (numbers) Total:	Nursing students, 2 year (12) (12)	Nursing students, 2 year (6) 3 year (6) (12)	B.supervisors (18) H.supervisors (7) (25)	Nursing students, 2 & 3 year (10) Patients (16) Supervisors (13) (39)	Patients (17) (17)	Students (34) Supervisors (38) Patients (33) (105)
Context	Psychiatric ward	Surgical and medical ward	Psychiatric, surgical and medical wardsomatic	surgical and medical ward	Surgical and medical ward	Psychiatric, surgical and medical ward
Aim of the study	to describe the learning process of students, in an encounter with patients, when supported by supervision given to pairs of students	to describe the learning process of student nurses towards their professional role when supported by supervision in pairs	to describe how supervisors support nursing students´ learning in pairs during their clinical practice	to explain and understand the learning space that occurs in the interaction between patient, pairs of nursing students and supervisors	to describe how patients experience being cared for by pairs of student nurses	
Data gathering Total:	Interviews in pairs (6) Diary entries (6) (12)	Interviews in pairs (6) (6)	Group interviews (4) Individual interviews (7) (11)	Observations (42) Interviews (individual and in pairs) (51) (93)	Individual interviews (17) (17)	Interviews (91) Diary entries (6) Observation (42) (139)
Analysis method	Phenomenologic analysis, RLR	Phenomenologic analysis, RLR	Phenomenologic analysis, RLR	Hermeneutic analysis, RLR	Phenomenologic analysis, RLR	

Note: The five included articles that form the general structure, showing the number of participants and data collection in each study and in the last column included participants and data collection in total.

### Methodological foundation in ReflectiveLifeworld Research (RLR)

Studies using RLR with their essential level of abstraction can contribute to a new understanding when essences of two or more studies are analysed together. A reflective lifeworld research approach is suitable as a theoretical foundation in order to do this. RLR is based on the concept of Husserl’s theories of the lifeworld and intentionality. Research based on the lifeworld theory involves a reflective approach to understand and describe the meanings of phenomena. RLR contains the methodological principles of openness, flexibility and bridling. Openness and flexibility are needed to understand the phenomenon in a new way. Bridling is described as adopting a reflective approach in order to not understand the new phenomenon too quickly and instead slowdown the process of understanding and not being to quick about defining what is indefinite. The reflective approach is important regardless of whether the research is empirical or theoretical (Dahlberg et al., 2008). Derived from RLR, Lindberg et al. (2016) describe how a general structure can be created consisting of two or more empirical results i.e., essences (essential structures of meanings) on an essential level of abstraction. The analysis in the creation of a general structure can be described in terms of “figure and background”. A greater understanding of the phenomenon in focus can be attained by using “figure and background”, where meanings from one of the essences is placed as a figure that can be seen to stand out against a background, the meanings from the other essences and vice versa. The intention is to find new intertwined patterns of meanings of the studied phenomenon by attempting to understand the primary results in relation to each other (Lindberg et al., 2016).

### Analysis - development of a general structure

The analysis in the present study is based on the description of methodological support and analysis for a general structure of empirical findings based on the RLR approach inLindberg et al. (2016). The aim of the analysis was to describe the phenomenon “caring-learning space” based on the perspectives of patients, students and supervisors. A further aim of the analysis was to develop a general structure of the “caring-learning space” based on the five empirical results, i.e., the essential level of abstraction. The learning space was the focus in the initial phase of the analysis, but while reading the essences we began to realize the learning was highly intertwined with caring. The results from these five empirical studies thus raised new questions about the interaction between learning and caring from the perspective of patients, students and supervisors, and an interest in understanding how they relate to each other.

The analysis then continued by reading the essential meanings of the five empirical studies with an open attitude and guided by questions such as, *“How is the learning space related to caring?”* and *“How is the caring related to learning?”* and *“How are the caring and learning intertwined?”* The movement between figure and background was carried out by seeking how the essences related to each other on an abstract level of understanding. The general structure emerged by placing meanings from one of the essences as a figure with meanings from the other essences as a background, and vice versa. Openness, flexibility and bridling were used actively throughout the analysis to understand the new phenomenon but at the same time slowdown the process and not ascribe meanings to the phenomenon. New, intertwined patterns of meanings emerged as a “caring-learning” space.

#### Settings and didactic model for the empirical studies

The five phenomenological empirical studies (Holst & Hörberg, 2012,2013; Holst et al., 2017a, 2017b; Strömwall et al., 2018) that form the foundation for the general structure were carried out in a learning environment based on the model of Developing and Learning Care Units (DLCU) in general hospitals and psychiatric clinics in Sweden. DLCU are based on caring science didactics with a lifeworld perspective (Dahlberg & Ekebergh, 2008), which entails a reflective supervisory approach in order to bridge the gap between theory and practice and learning in pairs of students. Didactics grounded on the lifeworld theory means supporting the student based on their individual understanding, knowledge and experiences. Similarly, caring from a lifeworld perspective entails understanding the patient as a unique subject with memories, perspective and understanding from previous experiences and future expectations (Dahlberg & Ekebergh, 2008; Ekebergh et al., 2018; Ekebergh, 2011). A supervisory approach based on the lifeworld theory entails supporting the students based on a sensitive and reflective attitude and problematizing the patient situation in a dialogue, which encourages the students to understand and reflect over the patients’ narratives and situations. The pairs of students consist of one student from the second year and one student from third (final) year. The students collaborate during their five-week clinical practice, and care initially for one patient together and as they develop, they care for an increased number of patients. By the end of their clinical practice, each student is caring for her/his patients on their own with support from their fellow student and supervisors. A team of supervisors support the students’ learning. A head supervisor is responsible for the nursing students’ practical caring science perspective and learning assessment. A base supervisor supports students’ learning in the bedside area. A clinical lecturer (from the nursing education) contributes with the theoretical caring science perspective (Hörberg et al., 2014,2019).

#### Empirical foundation - A short summary of the five studies

The general structure is based on the results from the five empirical studies that are further described below, where the phenomena from each of the five studies form the headings, together with a short summary of the four essences and one interpretation.

I The learning process of students, in an encounter with a patient, when supported by supervision given to pairs of students

To be supervised in a pair of students means to meet and care for patients together. Students’ learning process in meeting patients seems to be difficult to discern and grasp in an unreflective form, but the experience that meeting patients creates seems to be important for moments of reflection and to achieve a learning process. Supervision is necessary to gain a deeper understanding of a patient’s lifeworld, which also enables processing experiences, where their own and others’ beliefs and understanding are problematized and where one’s caring style has room to grow and develop. The learning environment affects the students’ learning process. The involvement of staff, supervision and security in a pair of students is important (Holst & Hörberg, 2012).

II The learning process of nursing students towards their profession when supported by supervision in pairs

A space for learning is created both in terms of time and place and in the interaction with patients, supervisors and fellow students. Furthermore, the phenomenon is characterized by a balanced responsibility, openness, flexibility in response between student and supervisor and a structured learning environment. The cooperation that takes place within each pair of students demands reciprocity where each student gives and takes and where their learning process can both develop and get space. Reflection in a favourable atmosphere provides opportunities for the student nurses to reveal their weaknesses/their vulnerability and are supported and helped in moving on in the learning process (Holst & Hörberg, 2013).

III Supporting students’ learning in pairs

The support is characterized by being available for the students with a supervisory approach that is guided by reflection. A stable learning environment that has a sound theoretical foundation and structure is required in order to create conditions for students’ learning in pairs based on “learning together”, where the individual student is also reached and seen. These opportunities could be described as learning spaces that encompass both interpersonal encounters and caring situations. An exchange of knowledge and experience in the interplay between the pair of students and the supervisor, which is supported by reflection, occurs in these learning spaces. The structured and reflective supervisory approach for supporting learning together is challenged by the competing reality of the caring situation. The challenge entails being torn between giving optimal support to the pair of students and meeting the needs for giving care in the reality of the caring situation (Holst et al., 2017a).

IIII The learning space—interpersonal interactions between nursing students, patients and supervisors

An interpersonal linkage between the patient, the students, and the supervisor provides space for interpersonal movements. An ability to show respect and to take responsibility balances these movements and creates reversibility between cooperation and independence. The interpersonal movements are more or less dynamic and are dependent on the students’ and supervisors’ awareness of the others and their ability to take responsibility within the learning space. A learning space based on mutual respect creates the prerequisites for beneficial and supportive relationships. A supportive cooperation is, on the other hand, based on interpersonal relationships with dynamic movements, which strengthens the interpersonal linkages between patient, students, and supervisor and contributes to genuine care and learning. A favourable learning space is thus constituted from the movements between getting, giving, and taking some place as a prerequisite for optimal learning. Due to the inevitable interpersonal connections between the parties and the fact that the patients are the hub of the learning space, they find themselves in a position of dependency where they are unable to defend themselves from the learning space (Holst et al., 2017b).

V To be cared for by student nurses, supervised in pairs

Being involved in the students’ learning and being met with responsibility and a willingness to care and learn. This means being made the centre of attention, being seen, taken seriously and being listened to as a valuable human being, which is shown in respectful encounters characterized by, thoughtfulness, commitment and presence, and where needs are met. A sense of security emerges when the students’ care is of good quality. The students’ care is shown to be more flexible and has a more open approach. This is connected to the students openly sharing their knowledge, information and communication with each other, and with the patient, in a way that can be easily understood. The students’ openness and patients’ confidence in the care being provided under supervision contributes to the patients feeling secure and satisfied in the caring situation (Strömwall et al., 2018).

### Results—general structure

#### The “caring-learning space”– a general structure

The “caring-learning space” shows itself in terms of interpersonal relationships between patients, students, supervisors and other actors in the care environment. The meeting and the intertwining between the care environment/the caring space and the learning space take place in the interpersonal relationships that are created. It is only when all the actors in and around the learning space accept learning as a part of the caring, that caring and learning can be intertwined to form a “caring-learning space”.

The ethos of the “caring-learning space” originates in the patient’s situation, where a reflecting, caring approach provides possibilities for being attentive to his/her needs and regulates how the space is apportioned between the actors. The patient’s possibilities of being able to take his/her place and make his/her voice heard in the “caring-learning space” is crucial for the creation of an atmosphere that is based on security, receptivity and flexibility. The “caring-learning space” reveals its vulnerability and demands that responsibility is taken for both the caring and learning actions simultaneously, and that a balance is created between them. A mutual responsibility needs to be taken so that both caring and learning are provided with space and that the prerequisites exist for developing, which is based on there being a sense of security between the actors in the “caring-learning space”. The responsibility is taken by an interaction between the structure and the flexibility, where place is provided for both caring and learning with the possibility of co-existing in a “caring-learning space”.

Caring and learning have the possibility of co-existing when an interaction occurs between them, both relationally and in caring structures. Learning can be in the foreground in relation to caring as a background and vice versa. The interaction and the movement, which occurs between caring and learning, is reflected through the interpersonal relationships. A flexibility and a receptivity are seen where the learning is allowed to become visible and be integrated in the caring processes when caring and learning interact. The relational interaction and the care environment are thus important for creating place for both the caring and the learning and for these being able to co-exist in a “caring-learning space”.

The positive co-operation shows itself through a movement between caring structures and a receptivity for the possibilities for learning. It becomes optimal when a flexible “caring-learning space” is created that expands and contracts in different directions depending on the need for support in caring and learning. It is, however, the care needs that regulate how prominent the “caring-learning space” should be.

### Discussion/Reflection

The aim was to describe and gain a greater understanding of the phenomenon “caring-learning space” based on the perspectives of patients, nursing students, and supervisors in clinical practice contexts.

Reflection on the method

The five empirical studies and the generating of the general structure of this study are based on a RLR approach. An analysis inspired by RLR entails being open and bridling throughout the analysis. Such an approach entails understanding in a reflective way and slowing down the process of understanding (Dahlberg et al., 2008). Further understanding of the phenomena in focus, a “caring-learning space” was guided by questions related to the five empirical studies, which required an open and bridling approach to understand the primary research in each study in relation to each other and to merge the results onto a new and more abstract level. Moreover, a general structure has similarities to literature reviews as they are based on several empirical studies, although the former is based on studies using the same methodology and theoretical underpinnings. Furthermore a general structure can also contribute to a more abstract understanding of the phenomena, while a literature review generates an understanding of similarities and differences among the empirical studies. Another possibility to achieve the aim of the study would be to use a meta-synthesis but there is a risk of losing depth and transferability with such an approach.

A strength in this study is the access to the raw data for performing a phenomenological analysis and that the abstract level of the phenomenon contributes to a deeper understanding of the phenomenon. On the other hand, the abstract level of the general structure can lead to limitations when attempting to convert the theoretical knowledge into practice. A limitation in this general structure in terms of generalization is that the phenomenon has been studied in a specific context, DLCU, which may affect how the phenomenon appears and which may differ in other contexts. However, the DLCU model can contribute inspiring developments in clinical practice without an anchor in a theoretical perspective.

A general structure can be generalized to similar phenomena and contexts (Lindberg et al., 2016). The importance of forming a general structure is the development of a greater understanding of a complex phenomenon that can be understood on a more abstract level. It becomes obvious after the completion of five empirical studies focusing on understanding the experiences of students caring and learning in pairs of patients, students and supervisors that a more complex phenomenon could be discerned from their results. A more abstract understanding of the “caring-learning space” contributes to the development of how we perceive students’ learning in clinical practice and their contribution to patient-centred care, based on a lifeworld perspective.

### Reflections on the result

The general structure of the “caring-learning space” shows that caring and learning have the possibility of co-existing when an interaction and movement occurs between them, which is reflected through the interpersonal relationships between patients, students, supervisors and other healthcare professionals. A flexibility and a receptivity occurs when learning is allowed to become visible and integrated in the caring processes. Lindberg and Ekebergh, (2020), Hörberg et al. (2019) and Ekebergh et al. (2018) have indicated fruitful educational strategies for combining caring and learning in a reflective manner and for presenting a way of supporting this issue by a guiding foundation based on Husserl´s lifeworld theory. Students and carers can be developed in both caring and learning guided by lifeworld-led didactics, when the learners’ lifeworld is taken into consideration in the same way as the patients’ lifeworlds are taken into consideration in caring (Hörberg et al., 2019). Similarly, Ekebergh et al. (2018) describe an interaction between caring and learning, that unites them through the patient and the student getting involved in each other’s worlds, the patient’s world is shared by the illness and the student’s world consists of longing for understanding the patient’s world. Sandvik and Hilli (2022) also emphasize the value of an understanding-based education and the importance of reflective, critical thinking in a more meaningful and authentic approach rather than drilling students on particular skills. An example is described by Jaastad et al. (2022) who emphasize how reflection intertwines caring theory, experiences and pre-understanding, which enabled the students to gain a deeper understanding of the patient’s lifeworld and their own vulnerability and uniqueness. To enable the creation of an understanding-based “caring-learning space”, which can provide space for reflection to create a deeper understanding of the caring of patients and students’ learning and their relationship, a lifeworld perspective is fundamental. A secure, receptible and flexible “caring-learning space”, which is supported by a reflective supervising approach representing the lifeworld theory is needed.

Furthermore, the result demonstrates how the ethos of the “caring-learning” space originates in the patient’s situation, where a reflecting, caring approach provides possibilities for being attentive to and understanding her/his needs. It is also shown how the space is apportioned between the actors. Eriksson (2010) have described the ethos as an ethical approach and how it constitutes a foundation for all that is caring. A sense of becoming a nurse and developing an increased understanding of ethical awareness has been highlighted by Sandvik et al. (2014) in the encounters between students, patients and supervisors. An ethical awareness can be developed during the education as the students’ level of knowledge and understanding increases. Furthermore, the general structure presented in this study shows how nursing students and supervisors need to be attentive to the vulnerable situation of the patients because of being available for students learning by a reflective approach. The patient can be present and be heard in the “caring-learning space”, which is based on security, receptivity and flexibility. An example of how ethos can be seen in the “caring-learning space” is presented by Andersson et al. (2020) who describes patients being cared for by nursing students in a psychiatric context where they meet in a mutual process and in which patients get the opportunity to talk about their illness and vulnerability, while experiencing a sense of well-being. The students need to listen to the patients’ narratives to acquire lived experiences in the learning process. The patients have the courage to show their vulnerability in the mutual process, while the student gets the opportunity to understand and learn. Furthermore, the ethos of the “caring-learning space” is created when patients and students accept learning as a part of caring and caring as a part of learning. The “caring-learning space” becomes flexible by expanding and contracting in different directions depending on the need for support in the “caring-learning space”. Furthermore, to achieve this, it is suggested that the patient should be made visible as a co-creative actor who needs help, and through their co-creation a flexible “caring-learning space” is created.

## Conclusions and implications

In conclusion, a caring-learning space is established by the patient being the focal point, but also a co-creator in caring as well as learning regardless of her/his health status. This implies that the ”caring-learning space” exists when patients, students, supervisors and other healthcare professionals interact based on their ability and role in the space.

## Clinical and educational implications

- In order for the”caring-learning space” to be established, it is assumed that ethos permeates understanding and actions in clinical practice.
- The patient must be seen as a active co-creator in learning within the”caring-learning space”
- In a”caring-learning space” the conditions for interaction that are caring and learning at the same time are provided and which enables an interaction that develops and raises the quality of patient care, students’ learning and supervisors support.
